# Re-evaluation of prophylactic cranial irradiation in limited-stage small cell lung cancer: a propensity score matched analysis

**DOI:** 10.1093/jrr/rrab053

**Published:** 2021-07-06

**Authors:** Yuko Inoue, Kayoko Tsujino, Nor Shazrina Sulaiman, Mitsuru Marudai, Akifumi Kajihara, Shuichiro Miyazaki, Shuhei Sekii, Haruka Uezono, Yousuke Ota, Toshinori Soejima

**Affiliations:** Department of Radiation Oncology, Hyogo Cancer Center, Akashi City, Hyogo 673-8558, Japan; Department of Radiation Oncology, Hyogo Cancer Center, Akashi City, Hyogo 673-8558, Japan; Department of Radiation Oncology, Hyogo Cancer Center, Akashi City, Hyogo 673-8558, Japan; Department of Radiation Oncology, Hyogo Cancer Center, Akashi City, Hyogo 673-8558, Japan; Department of Radiation Oncology, Hyogo Cancer Center, Akashi City, Hyogo 673-8558, Japan; Department of Radiation Oncology, Hyogo Cancer Center, Akashi City, Hyogo 673-8558, Japan; Department of Radiation Oncology, Hyogo Cancer Center, Akashi City, Hyogo 673-8558, Japan; Department of Radiation Oncology, Hyogo Cancer Center, Akashi City, Hyogo 673-8558, Japan; Department of Radiation Oncology, Hyogo Cancer Center, Akashi City, Hyogo 673-8558, Japan; Department of Radiation Oncology, Hyogo Cancer Center, Akashi City, Hyogo 673-8558, Japan

**Keywords:** prophylactic cranial irradiation (PCI), small cell lung cancer (SCLC), limited stage small cell lung cancer (LS-SCLC), brain metastasis (BM)

## Abstract

We attempted to re-evaluate the efficacy of prophylactic cranial irradiation (PCI) in limited-stage small cell lung cancer (LS-SCLC) with more recent data. A total of 179 patients with LS-SCLC received radical thoracic radiotherapy and chemotherapy at our institution between 1998 and 2018. One hundred twenty-eight patients who achieved complete response (CR), good partial response (PR), and PR without progression for at least for one year after initial therapy were enrolled in this study. These patients were divided into a PCI group (group A, n = 43), and a non-PCI group (group B, n = 85). Survival outcomes were retrospectively evaluated. Because several background factors differed significantly between groups A and B, propensity score (PS) matching was performed as 1:1 match of the two groups. Finally, we analyzed 64 patients (group A/B = 32/32). Median follow-up periods were 53 and 31 months in groups A and B, respectively. There were no significant differences between the groups’ backgrounds. Two-year overall survival (OS) rates were 77% in group A and 62% in group B (p = 0.224). Two-year brain metastasis free survival (BMFS) rates were 85% in group A and 57% in group B (p = 0.008). The number of patients who underwent a brain imaging test for confirmation of no brain metastasis (BM) after radical thoracic radiotherapy and chemotherapy (before PCI) was 84 (group A/B = 32/52). A PS matched analysis for cases of pre-PCI brain imaging group, two-year OS rates for group A/B were 73/59% (p = 0.446). Two-year BMFS rates for group A/B were 91/52% (p = 0.021). Retrospectively, PS matched analysis revealed that adding PCI to LS-SCLC patients who achieved good thoracic control significantly improved BMFS, but OS did not improve.

## INTRODUCTION

Small cell lung cancer (SCLC) is known to have a poor prognosis due to rapid growth and early distant and loco-regional dissemination [1]. It accounts for 15–20% of all lung cancers [2]. Limited stage SCLC (LS-SCLC) was defined in the following way: the entire lesions were localized in the ipsilateral thorax and could be safely contained within a single radiation port. Median survival time of LS-SCLC was reported as 16–20 months with a five-year survival rate of 10–20% [1].

Over 10% of SCLC patients have brain metastases at initial diagnosis [1]. The effectiveness of initial chemoradiotherapy (CRT) for brain metastasis (BM) is limited because most cytotoxic drugs do not cross the blood–brain barrier [3], and the brain is a frequent site of relapse, even if initial therapy shows a good effect [2]. Brain metastases occur in more than 50% of two-year survivors who received radical thoracic radiotherapy [3]. Approximately 65% of patients have detectable BM on autopsy [2].

Several clinical studies were performed to evaluate prophylactic cranial radiation (PCI) in SCLC, and many trials showed that PCI is effective in reducing BM [3, 4]. A- meta-analysis showed that PCI improves not only brain metastasis free survival (BMFS) but also overall survival (OS) in patients with LS-SCLC [5]. In the latest version of the NCCN guidelines (2019), PCI is recommended for patients who had a good response to initial therapy (category I). However, the above recommendation is based on data from an era when brain imaging after thoracic CRT was not a standard of care.

Recently, the effectiveness of PCI for extensive stage SCLC (ES-SCLC) has caused controversy. The European Organization for Research and Treatment of Cancer (EORTC) trial in 2007 showed that PCI improves not only BMFS, but also OS similar to ES-SCLC [6]. However, another randomized trial, mainly including patients with ES-SCLC who underwent a brain magnetic resonance imaging (MRI) after thoracic CRT in Japan, showed that PCI did not contribute to improving OS in ES-SCLC patients [7].

Treatment strategies for LS-SCLC has been changing over recent decades. Brain MRIs were introduced into clinical practice since Manapov *et al*. reported that 13/40 (32.5%) patients who achieved complete response (CR) with thoracic CRT developed BM on MRI prior to PCI [8]. A combination of cisplatin (CDDP) and etoposide (ETP) became the first line chemotherapy regimen for LS-SCLC along with thoracic radiotherapy (RT) in the early 2000s. However, few reports are available on the re-evaluation of the efficacy of PCI in LS-SCLC [9–12]. However, we have analyzed the efficacy of PCI in treatment of LS-SCLC with more recent data from our institution.

## MATERIALS AND METHODS

### Patients

A total of 179 patients with LS-SCLC received radical thoracic radiotherapy and chemotherapy at our institution between 1998 and 2018. A pathological diagnosis was obtained from all patients, and a retrospective **c**hart review was conducted. To perform clinical staging, physical examination, thoracoabdominal chemotherapy (CT), brain MRI, and ^18^F-fluorodeoxyglucose positron emission tomography/CT (PET-CT) were performed. Patients were diagnosed by *UICC Classification* (7th edition). After initial therapy, all patients were re-staged with thoracoabdominal chemotherapy (CT) and brain MRI within four weeks after the final course of chemotherapy or radiotherapy sequentially. Tumor response was assessed as described in the Response Evaluation and Criteria in Solid Tumors. Of 179 patients, 51 were excluded from this analysis because of the effect of initial therapy, and more specifically stable disease (n = 3), partial response (PR) with progression within one year after initial therapy (n = 48). One hundred twenty-eight patients who achieved CR, good PR; response which is comparable with CR, and PR without progression for at least one year after initial therapy were enrolled in this study. Seventy-five patients achieved CR or good PR, and 53 patients achieved PR without progression for at least one year after initial therapy. There were two reasons why we included the PR cases without progression for at least one year after initial therapy for the current analysis. First, analysis for only CR and good PR cases did not provide reliable results because of the small number of cases. Second, the percentage of CR + good PR in our series was smaller when compared with other previous studies. The percentage of CR and good PR and PR cases were 42% and 57% in our series, respectively, but the percentage of CR was reported to be 50–60% in other previous studies. The possible reason for the larger proportion of PR cases in our study may be because we included some cases which should be determined as CR or good PR but were judged as PR. It may be related to the difficulty in evaluating the size of the remaining primary lesion accurately, based only on plain CT at the re-staging timing. We enrolled those in this study as a surrogate. The proportion of patients who underwent PCI for excluded PR groups (n = 48)/included PR groups (n = 53) was 14.5% and 16.9%, respectively. The proportion of patients who developed BM as a progression of their disease for excluded PR cases/included PR cases was 22.9% and 41.5%, respectively. Median time to detection of BM as a progression of their disease of excluded PR groups/included PR groups was 7.3 months (range: 2.1–15.6) and 7.2 months (range: 2.0–20.0), respectively. One hundred twenty-eight eligible patients were divided into a PCI group (group A, n = 43), and a non-PCI group (group B, n = 85). Survival outcomes were retrospective and the study participants provided written informed consent. The study protocol was approved by the Institutional Review Board of our institution (No. G-96).

### Treatment methods

As a definitive strategy, all patients received thoracic radiotherapy of at least 45 Gy. Tolerant patients received concurrent CRT, using accelerated hyper-fractionation consisting of 45 Gy in 30 fractions delivered twice daily over three weeks (n = 113). Six of 113 patients received late concurrent CRT because of the high-volume tumors that could be encompassed in a single radiation port at the first diagnosis. Those with higher-volume disease or poor performance status received radiotherapy sequentially after induction chemotherapy (n = 15). The irradiation field included the primary tumor, metastatic mediastinal/supraclavicular lymph nodes and the prophylactic mediastinal lymph node region in 127 of 128 patients. One patient who had a previous history of breast-conserving surgery with whole-breast irradiation underwent involved field irradiation for LS-SCLC. The primary tumor, metastatic mediastinal/supraclavicular lymph nodes and prophylactic nodal region, were irradiated using 6–10 MV photons with anterior–posterior opposed fields up to 30 Gy in 20 fractions twice daily. The primary tumor and metastatic mediastinal/supraclavicular lymph nodes were subsequently boosted up to 45 Gy using 6–10 MV photons, avoiding the spinal cord. Seventy-eight patients received CDDP or carboplatin (CBDCA)/etoposide (ETP) four cycles if tolerated. Twenty-eight patients received one cycle of CDDP/ETP or CBDCA/ETP concurrently with radiotherapy followed by three cycles of CDDP/irinotecan (CPT-11). Five patients received CDDP or CBDCA/CPT-11, followed by sequential radiotherapy. Three patients received CBDCA/vincristine/doxorubicin/ETP and four patients received ifosfamide/CDDP/ETP. Four patients received other regimens. Initial treatment response was evaluated using thoracoabdominal CT and brain MRI within four weeks after the final course of chemotherapy or radiotherapy. Adaptation of PCI was determined at the discretion of radiation oncologists and pulmonologists.

PCI was delivered at 25 Gy in 10 daily fractions, five days per week in 35 patients, 30 Gy/15 fr/3 w in six patients, 24 Gy/12 fr/3 w in one patient, and 30 Gy/10 fr/2 w in one patient. We adopted 25 Gy/10 fr/2 w as a standard strategy after Péchoux *et al.* reported increased mortality with dose escalated PCI in a randomized clinical trial that 25 Gy should remain the standard of care [13]. All cases were treated with 3-dimensional-conformal radiation therapy (3D-CRT) on the whole brain, using opposed lateral or left anterior oblique/right anterior oblique beams [2] using 6 MV photons.

### Follow-up

All the patients treated at our institution were generally followed by pulmonologists and radiation oncologists every four to six weeks in the first year and less frequently in subsequent years. Patients were followed for at least five years if they could regularly attend hospital appointments. A physical examination was performed at every visit. Generally, a CT and brain MRI were performed for the detection of recurrence in cases without symptoms. A CT was performed every two to three months, and a brain MRI was performed every three to four months especially a year after initial therapy. Some patients underwent PET-CT every year and others only when the tumor marker was elevated, or patients had some symptoms.

Treatment toxicity was evaluated according to Common Terminology Criteria for Adverse Events (CTCAE) version 4.0. Patients who received PCI underwent Mini Mental State Examination (MMSE) [14] before and after PCI to objectively evaluate neurocognitive function which was a late adverse event of concern. When the score of MMSE decreased by 3 or more points lower on the post-test than on pre-test, it was defined as impaired function. For the remaining patients who had not undergone MMSE both before and after PCI, we checked the description to indicate of cognitive decline in the medical records.

### Endpoints and statistical analysis

Endpoints of this study were OS and BMFS. OS was counted from the start date of the initial therapy to either the date of death from any cause or last follow-up for surviving patients. The BMFS was counted from the start date of the initial therapy to either the date of revealing BM or last follow up. Survivals were estimated by the Kaplan–Meier method and compared by the Log-Rank test. Multivariate analyses were used to identify significant prognostic factors for OS and BMFS by Cox proportional hazard model. A p-value less than 0.05 was statistically significant.

Because several background factors differed significantly between the PCI group (group A, n = 43) and non-PCI group (group B, n = 85), propensity score (PS) matching was performed as 1:1 match of the two groups. Finally, we analyzed 64 patients (group A/B = 32/32). SPSS 24 (IBM) was used for all the statistical analyses.

## RESULTS

One hundred twenty-eight patients were enrolled. Baseline characteristics are shown in Table 1. Of 75 patients who achieved CR or good PR, 34 patients received PCI (group A), and 41 patients did not receive PCI (group B). Those who did not receive PCI, despite evidence of CR or good PR after radical radiotherapy and chemotherapy, had poor performance status and comorbidity (n = 4) and made an informed choice (n = 4). Median PCI dose was 25 Gy (range: 24–30). PCI was delivered later, 34 days (range: 17–126) after the last chemotherapy cycle. Median interval of the start of any initial treatment PCI was 111 days (range: 57–227).

**Table 1:** Patients characteristics before and after PS matching

		Patients, No.							
		Before propensity score matching (128)				After propensity score matching (64)			
		PCI (n = 43)	No PCI (n = 85)	p value	HR(95%CI)	PCI (n = 32)	No PCI (n = 32)	p value	HR(95%CI)
Median follow up (months)		52.5	30.7			49.9	20.6		
background factor									
Age (y)	−69	22	38	0.357	0.703(0.337-1.468)	17	19	1	1.134(0.424-3.037)
	70-	21	47			15	13		
sex	Male	38	71	0.602	0.357(0.156-0.817)	28	25	0.509	1.960(0.512-7.298)
	Female	5	14			4	7		
performance status	0-1	42	81	0.663	0.482(0.052-4.452)	32	32	−	−
	2	1	4			0	0		
T stage	1-2	32	47	0.053	0.425(0.190-0.953)	24	22	0.782	0.733(0.245-2.192)
	3-4	11	38			8	10		
N stage	1-2	28	71	0.174	0.464(0.173-1.249)	26	27	1	1.246(0.339-4.588)
	3	15	14			6	5		
UICC stage	IIA-IIIA	32	46	0.013	1.499(0.502-4.477)	23	23	1	1.0(0.336-2.974)
	IIIB	10	39			9	9		
Concurrent chemotherapy	+	43	71	0.013	−	32	32	−	−
	−	0	14			0	0		
Accelerated hyperfractionated radiotherapy	+	43	70	0.002	−	32	32	−	−
	−	0	15			0	0		
Effect of initial therapy	CR,gPR	34	41	0.001	0.247(0.106-0.577)	23	22	1	0.861(0.294-2.519)
	PR without progression at least for 1 year after initial therapy	9	44			9	10		

Abbreviation: PCI, prophylactic cranial irradiation; T stage, tumor stage; N stage, nodal stage; CR, complete response; gPR, good partial response; OS, overall survival; BMFS, brain metastasis free survival; HR, hazard ratio

Two-year OS rates were 77.5% in group A and 59.2% in group B (p = 0.015). Two-year BMFS rates were 85.3% in group A and 56.6% in group B (p = 0.008). PCI was a significant prognostic factor for both BMFS and OS.

Several background factors differed significantly between groups as detailed below: clinical stage (T stage p = 0.053, UICC stage p = 0.013), use of concurrent chemotherapy (p = 0.003), use of accelerated hyperfractionated radiotherapy (p = 0.002), and response to initial therapy (p = 0.001). PS matching was performed as 1:1 match of the two groups. After adjustment for PS, 32 pairs were matched between the two groups.

**Fig. 1.**
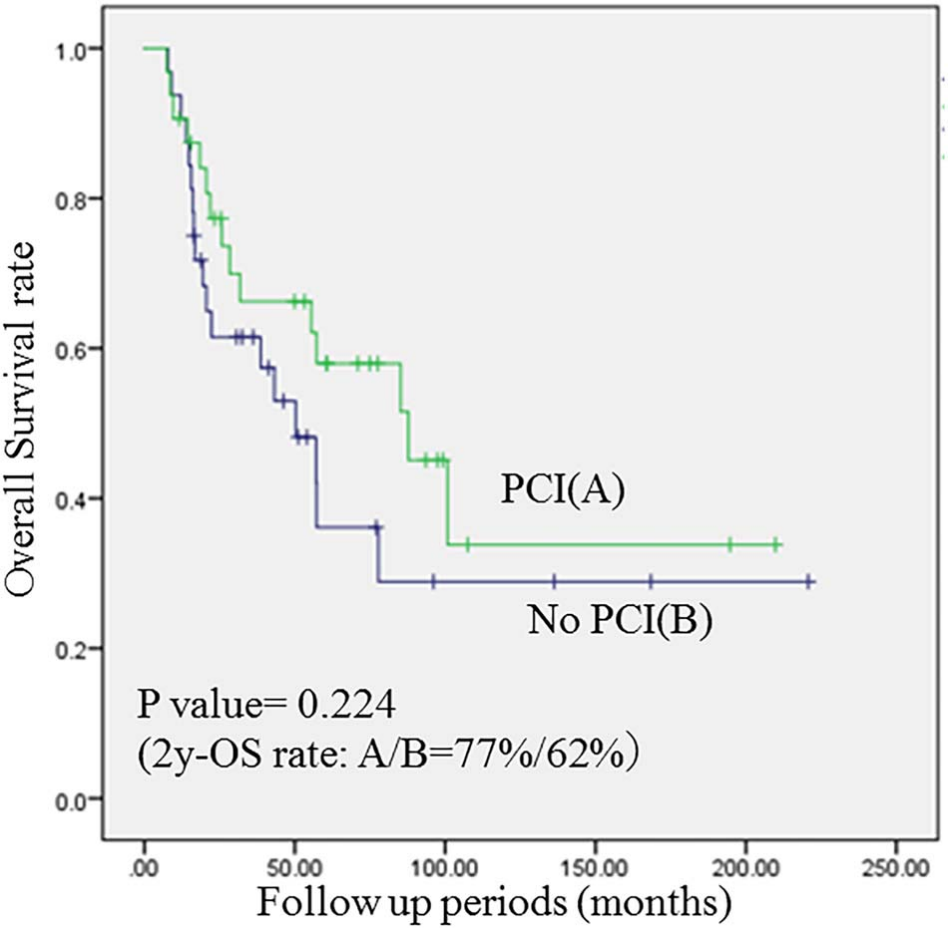
Comparison of OS rates in PS-matched patients who did and did not receive PCI. OS rates was not significantly different between the PCI and the non-PCI group.

The median follow up periods were 49.9 (range: 7.5–107.5) and 20.6 (range: 7.3–168.4) months in group A and B, respectively. There were no significant differences between the groups in backgrounds (age, sex, performance status, clinical stage, concurrent chemotherapy use, thoracic radiotherapy regimen and response to initial therapy). Lower nodal stage, UICC stage, and better response to initial therapy showed increased OS rates in univariate analysis. Additionally, nodal stage was a significant prognostic factor in multivariate analyses (Table 2). Two-year OS rates were 77% in group A and 62% in group B (p = 0.224) (Fig. 1).

**Table 2:** Univariate and multivariate analysis for OS and BMFS, after PS matching

Background factor		Patients, No.	OS			BMFS		
			univariate analysis	multivariate analysis		univariate analysis	multivariate analysis	
			p value	p value	HR(95%CI)	p value	p value	HR(95%CI)
Age (y)	−69	36	0.204	−	−	0.7	−	−
	70-	28						
Sex	Male	53	0.746	−	−	0.8	−	−
	Female	11						
Performance status	0-1	64	−	−	−	−	−	−
	2	0						
T stage	1-2	44	0.61			0.878	−	−
	3-4	18						
N stage	1-2	53	0.02	0.024	0.425(0.202-0.894)	0.305	−	−
	3	11						
UICC stage	IIA-IIIA	46	0.088	0.961	0.960(0.285-3.237)	0.533	−	−
	IIIB	18						
Concurrent chemotherapy	+	64	−	−	−	−	−	−
	−	0						
Accelerated hyperfractionated radiotherapy	+	64	−	−	−	−	−	−
	−	0						
Effect of initial therapy	CR,gPR	45	0.09	0.195	1.812(0.728-4.506)	0.32	−	−
	PR without progression at least for 1 year after initial therapy	19						
PCI	+	32	0.224	−	−	0.008	0.008	−
	−	32						

Abbreviation: PCI, prophylactic cranial irradiation; T stage, tumor stage; N stage, nodal stage; CR, complete response; gPR, good partial response; OS, overall survival; BMFS, brain metastasis free survival; HR, hazard ratio

**Fig. 2.**
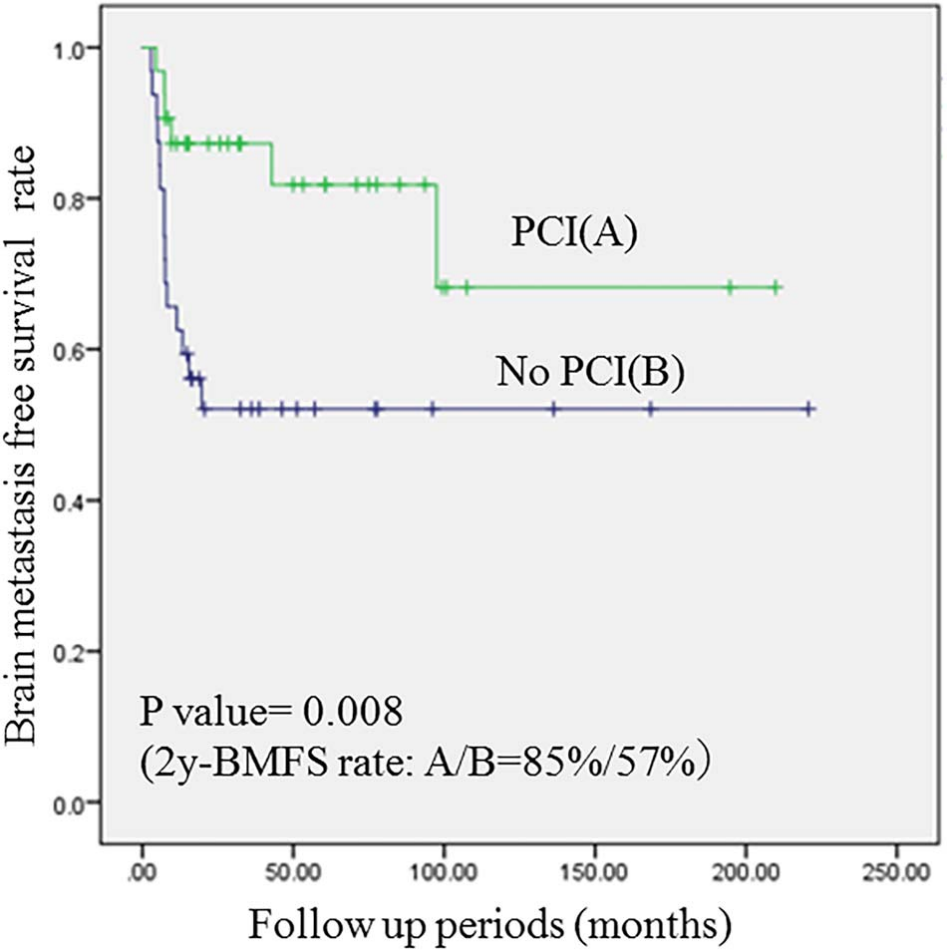
Comparison of BMFS rates in PS-matched patients who did and did not receive PCI. PCI significantly improved BMFS rates.

BMs were detected in 10 patients in group A, and 35 patients in group B by brain MRI. The median time to detection of BM was 12.9 months (range: 4.5–42.9) in group A and 7.5 months (range: 2–30.2) in group B (P = 0.003). PCI was the only significant prognostic factor for improved BMFS in multivariate analysis. Two-year BMFS rates were 85% in group A and 57% in group B (p = 0.008) (Fig. 2).

The number of patients who underwent brain MRI or CT to confirm absence of BM after thoracic (before PCI) was 84/128 (65.6%). Eighty-four patients were divided into group A (n = 32) and group B (n = 52). On a PS matched analysis for limiting cases from the pre-PCI brain imaging group, 23 pairs were matched between the two groups. Two-year OS rates for group A/B were 73/59% (p = 0.446). Two-year BMFS rates for group A/B were 91/52% (p = 0.021).

Ten patients from group A underwent MMSE both before and after PCI. The median follow-up period of MMSE from PCI was 13 months (range: 1–55). Impaired neurocognitive function was observed in five (15.6%) patients from group A. Four of five patients were diagnosed by MMSE, and another patient was diagnosed by medical records. One patient diagnosed by MMSE was not aware of their cognitive decline. For the remaining patients, there was no mention of cognitive decline in the medical records.

Grade 3 or greater late adverse events (taste disorders, otitis media, cataract and dermatitis) were not observed.

## DISCUSSION

In our study, PCI to LS-SCLC patients who achieved good thoracic control after initial therapy had significantly improved BMFS, but did not improve OS. The two-year OS rate was 77% in patients who received PCI (group A) and 62% in patients who did not receive PCI (group B), where there was no statistical difference. In the meantime, two-year BMFS rates were 85% in group A and 57% in group B, suggesting PCI may help to improve BMFS rates. In an analysis of patients who underwent brain imaging after initial CRT, we found the same results.

PCI had a significant positive impact on patient prognosis after radical treatment for LS-SCLC in the report from Nakamura *et al.* [11]. The differences between the PCI and non-PCI groups in terms of numbers and time to appearance of BM as a first recurrent site affected OS. These results were similar in all groups (pre-CRT MRI group/pre-PCI MRI group/pre-CRT and pre-PCI MRI group) [11]. Our study is similar in that PCI contributes to extending the time to appearance of BM.

Koh *et al.* released a report that stage I–II and a CR to initial therapy were good prognostic factors for BMFS and OS in the patient cohort who did not receive PCI, with those patients showing comparable survival outcomes with those who did receive PCI [12]. This study mentioned that PCI omission might be considered with reduced concerns in above cases, especially in complicated clinical situations where application of PCI is associated with concerns over comorbidities or neurotoxicity. PCI significantly improved BMFS but did not improve OS in our PS analysis, so we agreed that there could be some patient populations who would benefit relatively little from PCI.

Todd *et al.* concluded that in patients with LS-SCLC undergoing pre-PCI MRI, the use of PCI after radical thoracic radiation was not associated with a decrease in the risk of developing new BM. Additionally, the use of PCI was not associated with an OS benefit [10]. This study is comparable in using a PS matching analysis to evaluate the efficacy of PCI in LS-SCLC, but we obtained a different result in BMFS. A difference with populations, such as race or confounding factors considered in each study, might be a likely factor in different results.

To assess the validity of PCI, it is necessary not only to examine the effectiveness in the variant population but also to consider aspects of long-term adverse effects, especially chronic neurotoxicity. Common acute toxicity may include alopecia, headache, fatigue, nausea and vomiting, which are all manageable [5]. However, PCI may cause irreversible brain damage, and potentially lead to memory loss, intellectual impairment, dementia and ataxia as long-term effects [5]. In a previous retrospective study, 75% of patients receiving PCI showed impaired neurocognitive function manifesting as memory loss, ataxia and weakness [2]. In the Radiation Therapy Oncology Group (RTOG) 0212, approximately 62% of patients receiving 25 Gy of PCI showed chronic neurotoxicity. These reports showed the most significant risk factor was age [2,15]. In our study, impaired neurocognitive function was observed in 5 (15.6%) of 32 patients who received PCI. The median age of the five patients was 69 years, which was not significantly different from the whole of group A.

Chu *et al.* reported patterns of BM before PCI in LS-SCLC [16]. They conducted brain MRI in 110 LS-SCLC patients achieving CR after initial CRT and detected 129 pre-PCI intracranial lesions (24 patients). Of 129 pre-PCI intracranial lesions, two (1.5%) were in the hippocampal region. They reported the feasibility of hippocampus avoidance PCI to reduce neurotoxicity.

Indication for PCI should be carefully considered for its effectiveness given with possible adverse events. Moreover, we should consider the irradiated area to reduce long-term toxic effects compared with the risk of hippocampal recurrence when planning PCI.

This study has certain limitations; it had a retrospective design with a small number of patients.

However, we attempted to minimize the effect of potential confounders using PS matching to make an optimal evaluation of PCI. In addition, we believe this is the first study re-evaluating the efficacy of PCI in LS-SCLC using PS matching in predominantly Japanese patients.

In our retrospective study, PS matched analysis revealed that PCI significantly improved BMFS, but did not improve OS in patients with LS-SCLC who achieved a favorable response to thoracic CRT. Further prospective investigation with a larger cohort of patients to evaluate the validity of PCI in LS-SCLC in the era of modern treatment strategy is warranted.
